# A simplified memory network model based on pattern formations

**DOI:** 10.1038/srep07568

**Published:** 2014-12-19

**Authors:** Kesheng Xu, Xiyun Zhang, Chaoqing Wang, Zonghua Liu

**Affiliations:** 1 Department of Physics, East China Normal University, Shanghai, 200062, China

## Abstract

Many experiments have evidenced the transition with different time scales from short-term memory (STM) to long-term memory (LTM) in mammalian brains, while its theoretical understanding is still under debate. To understand its underlying mechanism, it has recently been shown that it is possible to have a long-period rhythmic synchronous firing in a scale-free network, provided the existence of both the high-degree hubs and the loops formed by low-degree nodes. We here present a simplified memory network model to show that the self-sustained synchronous firing can be observed even without these two necessary conditions. This simplified network consists of two loops of coupled excitable neurons with different synaptic conductance and with one node being the sensory neuron to receive an external stimulus signal. This model can be further used to show how the diversity of firing patterns can be selectively formed by varying the signal frequency, duration of the stimulus and network topology, which corresponds to the patterns of STM and LTM with different time scales. A theoretical analysis is presented to explain the underlying mechanism of firing patterns.

Understanding the brain is the most challenging problem in biology, neuroscience and network science. To figure out the underlying mechanisms, numerous experiments have been designed and several brain imaging techniques have been developed such as the computed tomography (CT), electroencephalography (EEG), positron emission tomography (PET), and functional magnetic resonance imaging (fMRI) etc^1^,^2^. By using these techniques, we now understand much better the events related to cognitive tasks and brain diseases such as seizure etc. Among all these events, memory is probably the one gotten the most attention so far.

It has been revealed that the process of memory has three sequence stages, from sensory memory(SM) to short-term memory (STM) and then to long-term memory (LTM)^3^,^4^,^5^,^6^,^7^,^8^. SM is brief, transient sensations, typically between 200 ms and 500 ms after the brain's cerebral cortex receives nerve information from ears, eyes and touch sensors. STM extracts messages from SM and can be maintained by active rehearsal and are easily displaced by new information or distractions. STM maintains information for a brief period before it is manipulated and is usually referred to as working memory^9^,^10^,^11^. The maintenance and manipulation of working memory is described as the executive control of working memory. STM may persist for minutes. LTM comes from STM and is maintained by more stable and permanent changes in neural connections widely spread throughout brain. LTM can last for hours, days, or even years.

Many areas of cortex are involved in STM (prefrontal, parietal, frontal eye fields), but hippocampus in brain is the key region for LTM, which receives information from cortex via multiple parallel pathways to each of its main subregions and is essential to transfer neural information from STM to LTM. When the hippocampus is damaged, the brain can not transfer new memories into LTM^12^. Under different behavior conditions, hippocampal networks show rhythmic oscillations in various frequency ranges such as theta (5–10 Hz) and gamma (30–100 Hz) frequency rhythms observed in the rat during exploration and rapid eye movement sleep^13^,^14^. It has been pointed out that different LTM correspond to different pattern dynamics in the brain network^13^,^15^,^16^,^17^, and interactions between neurons through synaptic links provide powerful computation capacities of the brain^18^,^19^. Further, it was revealed that the recurrent excitatory loops within a neural networks such as the cortico-striato-thalamo-cortical loop can sustain a persistent activity or pattern without external stimulus^20^,^21^.

To understand the mechanism of the above experimental findings, the coupled oscillator theory has been widely used to investigate the interaction among neurons. For example, it was pointed out that there is a large-scale cortical synchronization^22^,^23^ and synfire propagation in cognitive process where the signal is carried by a wave of synchronous neuronal activity within a subset of network neurons^14^,^24^,^25^,^26^. The corresponding theoretical studies have confirmed the solution of traveling waves in 1D and 2D medium^27^,^28^,^29^,^30^,^31^. Moreover, theoretical studies show that it is even possible to observe a localized states, called bump attractor in 1D lattice of coupled neurons, which is related to the working memory^32^,^33^. However, most cognitive functions are not represented in the brain by the activation of a single area but rather by a complex and rich behavior of brain networks distributed over various cortical and subcortical areas. Thus, it is necessary to extend the 1D and 2D lattices to a large network as information processing associated with higher brain functions is believed to be carried out by large scale neural networks^34^,^35^,^36^,^37^.

Most neural network models have assumed that synapses are placed among the neurons completely at random. However, experiments have shown that neural network is heterogeneous (some neurons have very many more synapses than others), clustered (two neurons have a higher chance of being connected if they share neighbors than if not) and highly modular (there are groups, or modules, with neurons forming synapses preferentially to those in the same module)^38^,^39^. Thus, recent studies usually assume that the functional brain networks have scale-free structures and focus on how the network topologies influence the network synchronization^40^. For further understanding how a large-size neural network processes temporal information through the dynamics of coupled neurons, a recent work shows the possibility of long-period rhythmic synchronous firing in a scale-free network where hub neurons are difficult to be activated and thus trigger synchronous firing across the network while loops formed by low-degree neurons determine the rhythm of synchronous firing^41^. That is, the existence of both the high-degree hubs and the loops formed by low-degree nodes are the two necessary conditions.

However, these theoretical studies discussed neither the transition from STM to LTM nor the diversity of the patterns of LTM. In this sense, an interesting question is how the diversity of the patterns of STM and LTM are generated in a network and how the patterns of STM are transferred into the patterns of LTM. To answer these questions, instead of considering a large network, we here present a simplified memory network model to represent the core of a brain function network, which consists of two loops of coupled excitable neurons with different synaptic conductance and with one node being the sensory neuron to receive an external stimulus signal. By this core network we show that the self-sustained synchronous firings can be observed even without the two necessary conditions in Ref. 41. Moreover, we find that this model may be used to show how the diversity of firing patterns can be selectively formed by varying the signal frequency, duration of the stimulus and network topology, which corresponds to the patterns of STM and LTM with different time scales. Based on this model, we also show that its extension to distributed synaptic conductance is possible. A theoretical analysis is presented to explain the diversity of firing patterns and the transition from STM to LTM.

## Results

### A simplified memory network model of coupled excitable neurons based on complex networks

**Figure 1:**
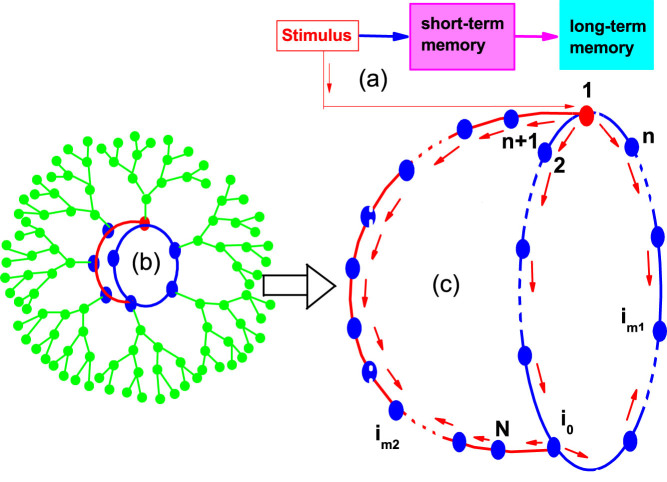
(a) Schematic figure of how a stimulus signal is first transformed to STM and then to LTM in a brain network. (b) A network of Cayley tree with a core where the blue and red nodes consist of the core part and the green nodes represent the grown surrounding nodes by an approach of Cayley tree. (c) A simplified memory network model consisting of *N* nodes with the nodes from 1 to *n* on a small closed loop and the nodes from *n* + 1 to *N* on a branch connected to the loop at the node *i*_0_. The node 1 is in charge of receiving external stimulus. The node *i*_0_ is shiftable so that the topology can be slightly changed to implement the diversity of memory patterns. The parameter *f* is chosen to be *f_b_* for the nodes from node 1 to node *n* and *f_r_* for the nodes from node *n* + 1 to node *N*, with *f_b_* < *f_r_*.

Figure 1(a) shows the schematic figure of how a stimulus signal is first transformed to STM and then to LTM in a brain network. This cognitive process usually involves several areas of cortex and is thus believed to be implemented in a large network such as the brain network. A characteristic feature of memory is that both STM and LTM can be considered as a variety of patterns where STM may last for a shorter time while LTM may last much longer time^13^,^15^,^42^. From the aspect of patterns, it is maybe convenient and important if we can grasp the characteristic feature of a large brain functional network by a core network with small size. For this purpose, we here present a simplified memory network model to implement the process from STM to LTM as shown in Fig. 1(c), which may come from a typical motif or core part of a large-size network such as the network of Cayley tree in Fig. 1 (b) where the blue and red nodes denote the core part and the green nodes represent the grown surrounding nodes by an approach of Cayley tree^43^. In detail, Fig. 1(c) consists of *N* nodes with the nodes from 1 to *n* on a small closed loop and the nodes from *n* + 1 to *N* on a branch connected to the loop at the node *i*_0_. The node 1 in Fig. 1(c) is in charge of receiving external stimulus. The node *i*_0_ is shiftable so that the topology can be slightly changed to implement the diversity of memory patterns. We here focus on whether a LTM pattern can be formed in this core network and how the intervals of firings influence the diversity of patterns.

To investigate the dynamics of the core network model of Fig. 1(c), we let each node represent an excitable neuron and the coupling be bidirectional. In this way, all neurons will be connected to their two neighbors except the neurons 1 and *i*_0_ connected to their three neighbors. For simplicity, we choose the external signal as a sinusoidal waveform *I_ext_* = *A* sin(*ωt*) + *B*, which will be detected by the sensory node 1. In detail, we let each node in Fig. 1(c) be an excitable FitzHugh-Nagumo (FHN) neuron^44^,^45^,^46^ and let the coupling be a chemical coupling. The dynamics of neurons can be described as 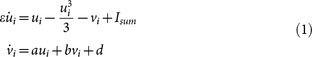
where *I_sum_* = *I_n_* + *I*_2_ + *I_n_*_+1_ + *I_ext_* for the sensory node 1, 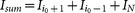 for the node *i*_0_, *I_sum_* = *I*_1_ + *I_n_*_+2_ for the node *n* + 1, *I_sum_* = *I*_1_ + *I_n_*_−1_ for the node *n*, 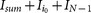 for the node *N*, and *I_sum_* = *I_i_*_+1_ + *I_i_*_−1_ for the other nodes. *u_i_* and *v_i_* represent the fast and slow variables, respectively. *ε* is a small parameter which warrants a clear separation between the slow and fast time scales. We fix the parameters *ε* = 0.01, *a* = 0.08, *b* = −0.064, and *d* = 0.056 as in Ref. 44, 47 so that an isolated neuron will be in the excitable state. The expression of each *I_i_* in *I_sum_* can be given as *I_i_* = *g_syn_*(*u_syn_* − *u_i_*) with 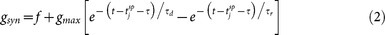
where *g_max_* describes the maximal synaptic conductance between neurons, *u_syn_* denotes the synaptic reversal potential, *τ* is the time delay between adjacent neurons, 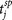 represents the presynaptic spiking, *τ_d_* and *τ_r_* stand for the decay and rise time of the function and determine the duration of the response^48^,^49^,^50^,^51^. The parameter *f* is a small quantity and represents the synaptic conductance of the open channels in steady state^1^,^47^,^49^,^50^. In this paper, we take the parameters as *g_max_* = 0.35 *mS*/*cm*^2^, *u_syn_* = 0, *τ* = 0.5 *ms*, *τ_d_* = 10 *ms*, *τ_r_* = 1 *ms*, *N* = 150 and *n* = 30.

For the case of identical *f* to all neurons, we find that there will be no firings in the excitable system of Eq. (1) when there is no external stimulus, i.e. *I_ext_* = 0. However, when an external stimulus of *I_ext_* > 0 is added, some firing patterns will be generated and can be considered as the patterns of STM. We further find that these firing patterns will quickly disappear once the external stimulus is switched off, see Fig. 1 in SI for details, indicating that a self-sustained pattern of LTM cannot be formed. The reason is that after each firing, a neuron will enter a refractory status for some time and thus results in a gap of no firing. From the arrows of Fig. 1(c) we see that there are two loops of firings propagation, i.e. a smaller loop 1 → 2 → *i*_0_ → *i_m_*_1_ → *n* → 1 and a larger loop 1 → 2 → *i*_0_ → *N* → *i_m_*_2_ → *n* + 1 → 1. In each loop, there are two propagation directions of firings and the firings from these two directions will meet at the middle node, i.e. *i_m_*_1_ = 16 in the smaller loop and *i_m_*_2_ = 95 in the larger loop. Once two firings are transmitted to the node *i_m_*_1_ (*i_m_*_2_) from its both left and right sides, its two neighboring nodes *i_m_*_1−1_ and *i_m_*_1+1_ (*i_m_*_2−1_ and *i_m_*_2+1_) will be in the refractory status and thus the two encountered firings at *i_m_*_1_ (*i_m_*_2_) cannot cross each other to make a further propagation, which results in an effect of annihilation at the node *i_m_*_1_ (*i_m_*_2_) and has been confirmed by Fig. 1(a) and (b) in SI.

To make the system of Fig. 1(c) show a LTM pattern, we let the parameter *f* be non-identical for each neuron in the rest part of this paper. This setting is based on the observation that there are many different kinds of neurons in cortical neural networks and they are nonidentical even for the ones in the same kind of neurons^1^,^2^,^9^,^51^. We here use *f* to reflect the nonidentity of neurons. For simplicity, we first let *f* take only two different constants, i.e. *f_b_* for the nodes from node 1 to node *n* and *f_r_* for the rest nodes, with *f_b_* < *f_r_*.

### Self-sustained patterns in the core network

**Figure 2:**
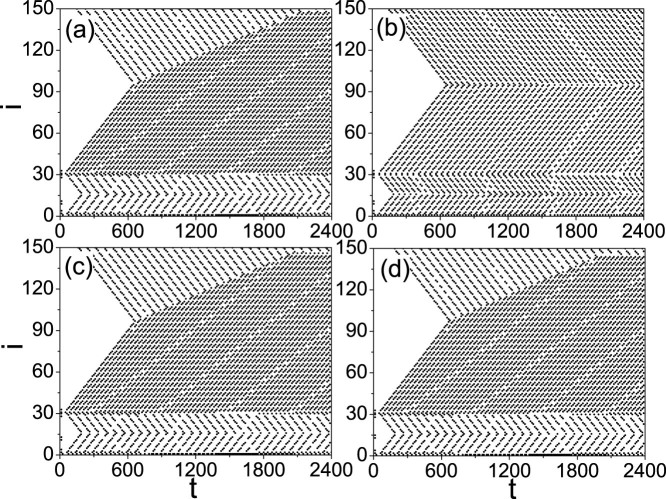
Cases of persistent stimulus with (a) *i*_0_ = 10, *ω* = 0.5; (b) *i*_0_ = 10, *ω* = 0.75; (c) *i*_0_ = 12, *ω* = 0.5; and (d) *i*_0_ = 14, *ω* = 0.5.

In numerical simulations, we fix *f_b_* = 0.031 and *f_r_* = 0.05 if without specific illustration. We first add an external stimulus *I_ext_* = *A* sin *ωt* + *B* with *A* = 1 and *B* = 1.2 to the source node 1 and calculate Eq. (1). Figure 2 shows the evolution of firings at each nodes where (a) represents the case of *i*_0_ = 10, *ω* = 0.5, (b) the case of *i*_0_ = 10, *ω* = 0.75, (c) the case of *i*_0_ = 12, *ω* = 0.5, and (d) the case of *i*_0_ = 14, *ω* = 0.5. From Fig. 2 we see that the firing patterns at the four panels are different each other, indicating that the firing patterns may be influenced by both the parameters *i*_0_ and *ω*.

**Figure 3:**
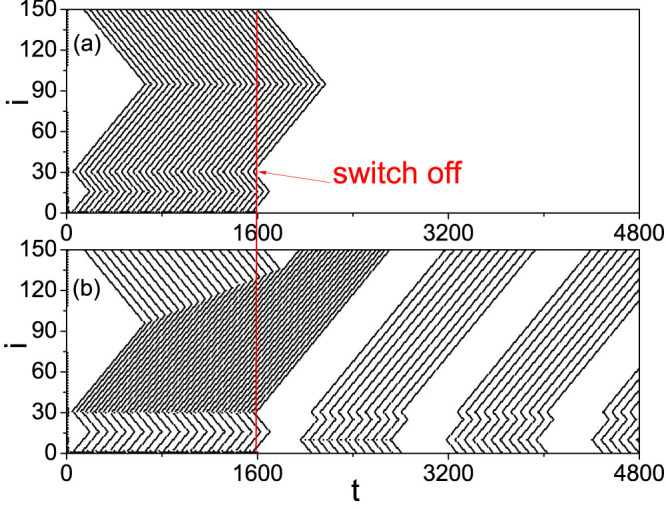
Two kinds of patterns after switching off the stimulus at *t* = *T*_0_ = 1600. (a) STM with *i*_0_ = 10, *ω* = 0.75 and (b) LTM with *i*_0_ = 10, *ω* = 0.5.

On the other hand, from Fig. 2 we observe that the firing patterns in panel (b) is regular while the other three panels are irregular. Is the regularity related to STM and LTM? To figure out the answer, we check the effect of switching off the external stimulus *I_ext_* at *t* = *T*_0_. By an ensemble of numerical simulations we find that after the switching off, there are two kinds of behaviors. One is the quick decaying until disappearance of the patterns and the other is self-sustained patterns. Figure 3(a) and (b) show two such examples for *T*_0_ = 1600, respectively, where (a) represents the case of decaying and (b) the case of self-sustained pattern. Noticing their different time scales, we consider the decaying patterns as STM and the self-sustained patterns as LTM.

Except the two parameters *i*_0_ and *ω*, our numerical simulations show that the duration of stimulus, *T*_0_, is another parameter to influence the firing patterns, i.e. STM and LTM. That is, for a set of fixed *i*_0_ and *ω*, the self-sustained patterns may be different for different *T*_0_, see Fig. 2 in SI for details. A common feature in the four panels of Fig. 2 in SI is that the patterns in each panel are periodic, indicating that we may define a quantity to represent the patterns. For this purpose, we introduce *S_p_* to represent the stabilized spiking number of a pattern in LTM, i.e. the spiking number in a period of repeating patterns. For example, we have *S_p_* = 11 Fig. 3(b) and *S_p_* = 2, 6, 8 and 10 in Fig. 2 (a)–(d) of SI, respectively.

**Figure 4:**
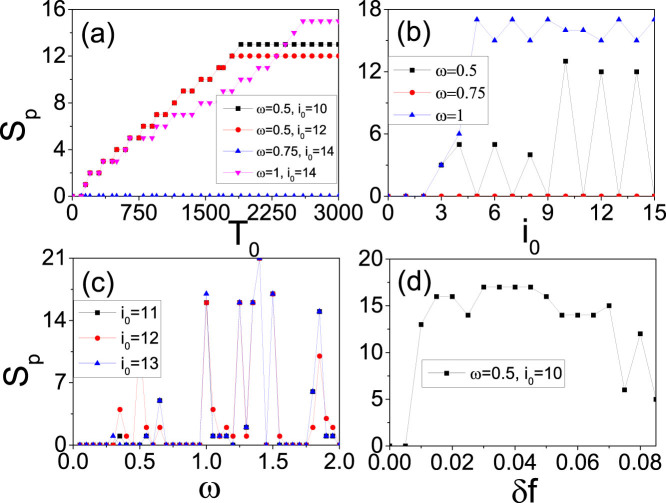
Dependence of the spiking number *S_p_* on the parameters, i.e. the duration of stimulus *T*_0_, the junction node *i*_0_, the signal frequency *ω* and the synaptic conductance difference *δ f*. (a) represents the case of *S_p_* on *T*_0_ for different pairs of *ω* and *i*_0_ where the four curves represent the cases of *ω* = 0.5 and *i*_0_ = 10, *ω* = 0.5 and *i*_0_ = 12, *ω* = 0.75 and *i*_0_ = 14, and *ω* = 1 and *i*_0_ = 14, respectively. (b) represents the case of saturated *S_p_* on *i*_0_ where the three curves represent the cases of *ω* = 0.5, 0.75, 1, respectively. (c) represents the case of saturated *S_p_* on *ω* where the three curves represent the cases of *i*_0_ = 11, 12, 13, respectively. (d) represents the dependence of saturated *S_p_* on *δ f* with *i*_0_ = 10, *ω* = 0.5 and *T*_0_ = 3000.

To figure out the relationship between *S_p_* and *T*_0_, Fig. 4(a) shows the dependence of *S_p_* on *T*_0_ for different pairs of *ω* and *i*_0_ where the four curves represent the cases of *ω* = 0.5 and *i*_0_ = 10, *ω* = 0.5 and *i*_0_ = 12, *ω* = 0.75 and *i*_0_ = 14, and *ω* = 1 and *i*_0_ = 14, respectively. From Fig. 4(a) we see that *S_p_* may be zero or increases with *T*_0_ gradually and then arrives a stabilized value in all the cases, indicating a saturation of *S_p_*. The existence of *S_p_* = 0 implies that the self-sustained patterns are not guaranteed for an arbitrary *ω* but formed only selectively. To figure out the dependence of *S_p_* on the parameters *i*_0_ and *ω*, we take a relatively larger *T*_0_ = 3000 so that *S_p_* will be stabilized. Fig. 4(b) and (c) show the dependence of the saturated *S_p_* on *i*_0_ and *ω*, respectively. From Fig. 4(b) and (c) we see that for fixed *ω* (*i*_0_), *S_p_* may be zero on some *i*_0_ (*ω*), confirming again that the formation of self-sustained LTM patterns are selectively. From Fig. 4(b) and (c) we also noticed that *S_p_* is nonlinear on both *ω* and *i*_0_, which can be understood as follows. Noticing that a signal in brain is usually detected by phase-locking^52^,^53^, we may assume that there is also phase-locking in LTM patterns. To confirm it, we have checked the time interval between two consecutive firings at a node in Fig. 2, which consists of the firing time and refractory time of a neuron. We interestingly find that the interval is about 50 for all the neurons in Fig. 2(b), indicating that a homogeneous phase-locking is formed. While the intervals in the other three panels of Fig. 2(a), (c) and (d) are the same and their intervals in the denser and sparse parts are about 37.5 and 75, respectively, indicating that the latter is double of the former and thus a 2:1 phase-locking is formed. Therefore, the homogeneous phase-locking will produce the case of *S_p_* = 0 in Fig. 4 while the heterogeneous phase-locking such as 2:1 phase-locking will produce the cases of *S_p_* > 0 in Fig. 4. The match between *i*_0_ and *ω* will determine the type of phase-locking, which results in the nonlinear increase in Fig. 4(b) and (c).

In sum, a diversity of firing patterns can be obtained by changing the three parameters *T*_0_, *i*_0_ and *ω*. This finding is helpful to understand the origin of STM and LTM patterns. Except the three key parameters *T*_0_, *i*_0_ and *ω*, we find that there is one more necessary condition for successfully observing LTM patterns, i.e. the synaptic conductance difference *δ f* ≡ *f_r_* − *f_b_*. The range of *δ f* for STM and LTM patterns may partially reflect the model performance^54^, i.e. whether the model produces the STM/LTM behavior through a wide range of parameter values, or only in the limited range of parameter values that correspond to human neural parameters. Fig. 4(d) shows the dependence of the saturated *S_p_* on *δ f* where the three key parameters are fixed at *i*_0_ = 10, *ω* = 0.5 and *T*_0_ = 3000. It is easy to see that *S_p_* depends on *δ f* and the valid range of *δ f* is limited, confirming again that *δ f* is the main reason to generate LTM. This finding tells us that the neurons for cognitive task must be nonidentical but their difference should not be too much, which is consistent with the small *f* assumption in Eq. (2).

### A case of extension to heterogeneous choice of *f*

**Figure 5:**
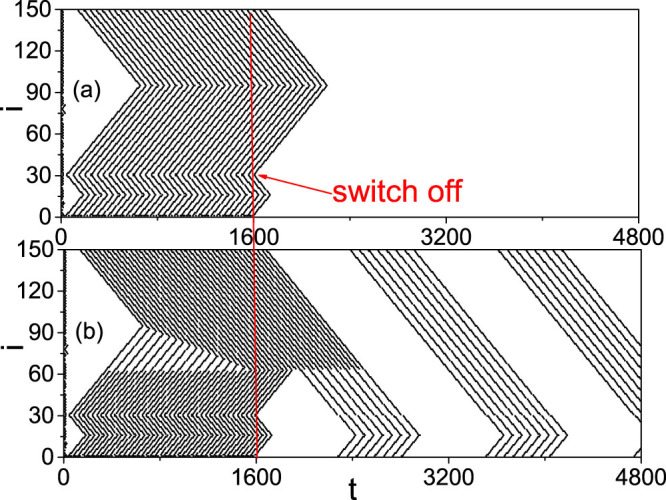
STM/LTM patterns after switching off the stimulus at *t* = *T*_0_ = 1600 for the case of distributed *f* with *E* = 0.06 and *σ* = 0.01, which corresponds to Fig. 3. (a) STM with *i*_0_ = 10, *ω* = 0.75 and (b) LTM with *i*_0_ = 10, *ω* = 0.5.

**Figure 6:**
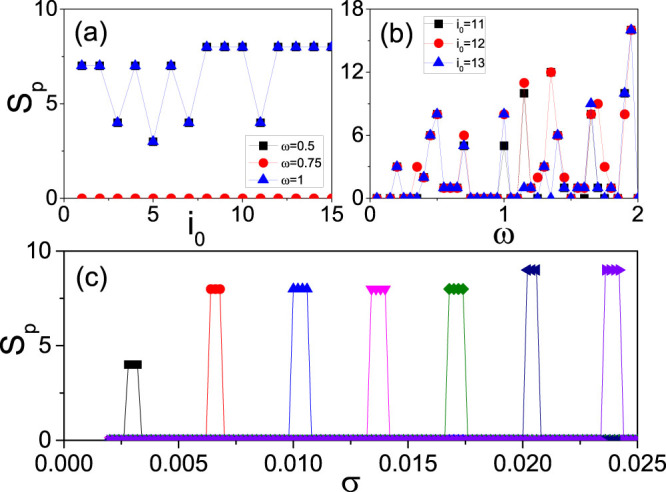
Dependence of *S_p_* on the parameters *i*_0_, *ω*, *E* and *σ* for the case of *f_b_* and *f_r_* taking the Gaussian distribution with average *E* and variance *σ*. The other parameters are taken as the same as in Fig. 4. (a) represents the case of saturated *S_p_* on *i*_0_ with *E* = 0.06 and *σ* = 0.01 where the three curves represent the cases of *ω* = 0.5, 0.75, 1, respectively. (b) represents the case of saturated *S_p_* on *ω* with *E* = 0.06 and *σ* = 0.01 where the three curves represent the cases of *i*_0_ = 11, 12, 13, respectively. (c) represents the dependence of saturated *S_p_* on *σ* with *i*_0_ = 10, *ω* = 0.5 and *T*_0_ = 3000 where the curves from left to right represent the cases of *E* = 0.04, 0.05, 0.06, 0.07, 0.08, 0.9 and 0.1, respectively.

In order to introduce more realism in the network as well as to remark the importance of *f* diversity, it could be useful to consider a totally heterogeneous distribution of *f*, instead of the two different constants of *f_b_* and *f_r_*. We here take the Gaussian distribution as an example, i.e. we let each neuron with a different value of f extracted from a Gaussian distribution with average *E* and variance *σ* and thus the two population of neurons are now with identical parameters. We interestingly find that both the STM and LTM patterns can be still observed. Fig. 5 shows the results for *E* = 0.06 and *σ* = 0.01, where the other parameters are remained the same as in Fig. 3. Comparing Fig. 5 with Fig. 3, we see that they are similar with each other, indicating that the STM and LTM patterns can be also produced from the distributed *f*. To confirm the diversity of patterns, Fig. 6(a) and (b) show how *S_p_* depends on *i*_0_ and *ω*, respectively. We see that they are similar to Fig. 4(b) and (c), respectively. Furthermore, we find that there is no *S_p_* when *E* < 0.04 and the system will have spontaneously firings when *E* > 0.1, indicating that the STM and LTM patterns can be only observed in the limited range of *E* ∈ [0.04, 0.1]. Fig. 6(c) shows how *S_p_* depends on *σ* for different *E*, where the peaks from left to right represent the cases of *E* = 0.04, 0.05, 0.06, 0.07, 0.08, 0.09 and 0.1, respectively. From Fig. 6(c) we see that the range of *σ* for each *E* is narrow, confirming again the limited range in Fig. 4(d).

**Figure 7:**
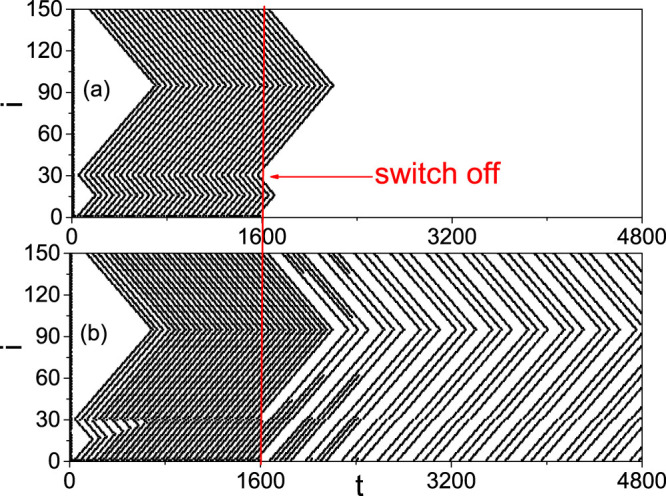
STM/LTM patterns after switching off the stimulus at *t* = *T*_0_ = 1600 for the case of distributed *g_max_* with *E* = 0.5 and *σ* = 0.04 and fixed *f_b_* = *f_r_* = 0.03, which corresponds to Fig. 3. (a) STM with *i*_0_ = 10, *ω* = 0.75 and (b) LTM with *i*_0_ = 10, *ω* = 0.5.

Instead of the distributed *f*, the non-identity of neurons may be also reflected by the parameter *g_max_*. In this sense, we find that the STM and LTM patterns can not be observed for the case of *f_b_* = *f_r_* = 0 but can be still observed if we choose a small *f_b_* = *f_r_* and a distributed *g_max_*, indicating that the *f* term is essential for the LTM dynamics. Fig. 7 shows the results for a Gaussian distribution of *g_max_* with average *E* = 0.5 and variance *σ* = 0.04, where *f_b_* = *f_r_* = 0.03 and the other parameters are remained the same as in Fig. 3. It is easy to see that Fig. 7 is similar to both Fig. 3 and Fig. 5. Thus, the STM and LTM patterns can be also produced by the distributed *g_max_*.

### A theoretical analysis

**Figure 8:**
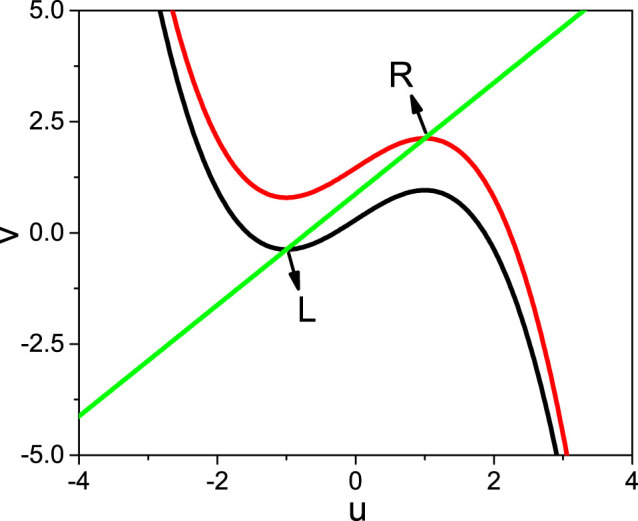
Solutions of the nullcline Eqs. (3) and (4) where *L* and *R* represent the two extreme points.

Through the core network model of Fig. 1(c) we have numerically observed the rich patterns of STM and LTM, which is usually observed in huge networks such as brain functional networks. This finding tells us that the complicated cognitive process may be recurred by a simple network topology, implying that the underlying mechanism may be very simple. Then, an interesting question is why such a simple system can do it or what is the mechanism behind the system. To answer this question, we first go back to the dynamics Eq. (1) at the source node 1. All the firing patterns are initially generated at this node, thus the condition for this source node to generate a firing is the key to both STM and LTM. Initially, the source node is equivalent to an isolated neuron with 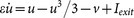, 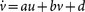. Its nullcline equation can be given as 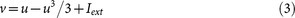

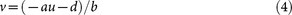
There are two extreme points *u* = ±1 in Eq. (3). Substituting them into Eq. (4) we obtain *v_L_* = −0.375 and *v_R_* = 2.125. Then substituting *v_L_*_,*R*_ back to Eq. (3) we obtain *I_L_* = 0.2917 and *I_R_* = 1.4583. Let *I_range_* = [*I_L_*, *I_R_*]. Figure 8 shows the solutions. Thus, the condition to generate a firing is *I_L_* < *I_ext_* < *I_R_*. This condition can be used to determine the parameters *A* and *B* in *I_ext_* = *A* sin *ωt* + *B* and can be also used to figure out the value of *f* in Eq. (2).

We have assumed that the system Eq. (1) is an excitable system and has no firing when there is no external stimulus, implying that *f* cannot be too large. Otherwise, *f* will directly induce a firing when there is no *I_ext_*. This restriction can be used to figure out the maximum *f_max_*. When there is no firing and no stimulus *I_ext_*, the source node 1 will get three couplings *I_n_*, *I*_2_ and *I_n_*_+1_ from its three neighbors with *I_i_* = *g_syn_*(*u_syn_* − *u_i_*) = −*fu_i_*. By the condition of no firing we have *I_n_* + *I*_2_ + *I_n_*_+1_ < *I_L_*, which gives approximately *I_i_* = −*fu_i_* < *I_L_*/3 = 0.0972. By numerical simulations we find *u_i_* ≈ −1.5 at the rest state, which gives *f_max_* = 0.0972/1.5 ≈ 0.065. That is, once the average *f* from the three neighbors of the source node 1 is over this *f_max_*, a spontaneous firing after switching *T*_0_ off will be still possible and it will thus influence *S_p_* randomly. This prediction has been confirmed by the big fluctuation of *S_p_* in Fig. 4(d) when *δ f* > 0.07. Take *δ f* = 0.08 as an example. We have *f_b_* + *f_b_* + *f_r_* = 0.031 + 0.031 + (0.08 + 0.031) = 0.173, which is close to 3 × 0.065 = 0.195 and thus can explain the big fluctuation. For fixed *f_b_* and *f_r_* with *f_b_* < *f_r_* < *f_max_*, there will be a firing when *I_n_* + *I*_2_ + *I_n_*_+1_ + *I_ext_* is located between *I_L_* and *I_R_*, which gives *B* > *I_L_* − 1.5(2*f_b_* + *f_r_*) for an arbitrary *A* or *A* > *I_L_* − 1.5(2*f_b_* + *f_r_*) − *B* for a given *B*. Obviously, the chosen parameters *A* = 1 and *B* = 1.2 in the above numerical simulations satisfy these conditions.

We now turn to study the transition from STM to LTM and aim to understand why some patterns of STM can be transformed into LTM while the others cannot. Take Fig. 2 as an example. From Fig. 4(b) we notice that the patterns in the panels (a), (c) and (d) of Fig. 2 will become self-sustained patterns when the stimulus signal is switched off at *T*_0_ = 3000, while the pattern in the panel (b) of Fig. 2 will disappear. The reason can be figured out by comparing the four panels of Fig. 2, which shows that the panel (b) of Fig. 2 has a homogeneous pattern while the other panels (a), (c) and (d) of Fig. 2 have heterogeneous patterns. As *f_b_* is different from *f_r_*, the branch of *f_b_* in Fig. 1(b) (the nodes of 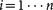) will have different refractory periods with the branch of *f_r_* (the nodes 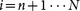). When the frequency *ω* of stimulus is matched with the refractory period of the branch of *f_b_*, it will not match with that of the branch of *f_r_*, and vice-versa. Thus, the density of firings for the branch of *f_b_* will be different from that of the branch of *f_r_*, confirmed the panels (a), (c) and (d) of Fig. 2. When the frequency *ω* is not matched with both the branch of *f_b_* and the branch of *f_r_*, the two branches may have the same density of firings, confirmed the panel (b) of Fig. 2. The Fig. 3 in SI shows a more detailed analysis on this aspect.

We first focus on the case of the panel (b) of Fig. 2, i.e. the case of a homogeneous pattern at both the branch of *f_b_* and the branch of *f_r_*. It will become Fig. 3(a) when the switch off is considered at *T*_0_ = 1600. From Fig. 3(a) we see that there are two minimum points *i_min_* = 16 and 95 in each “W” shaped curve for both before *t* < *T*_0_ = 1600 and after *t* > *T*_0_ = 1600. This result can be explained by the network topology of Fig. 1(c). When a firing is generated at the source node 1, it will be spread out along the two loops (see the red short arrows in Fig. 1(c)) through the three neighboring nodes 2, *n* and *n* + 1. In the smaller loop of 1 → 2 → *i*_0_ → *i_m_*_1_ → *n* → 1, the two firing waves from the nodes 2 and *n*, respectively, will meet at the node *i_m_*_1_ (see the arrows there) and then cancel each other because both its two neighbors, i.e. *i_m_*_1_ → 1 and *i_m_*_1_ + 1, will be in the refractory status at this moment and thus cannot immediately fire again. Similarly, in the larger loop of 1 → 2 → *i*_0_ → *N* → *i_m_*_2_ → *n* + 1 → 1, the two firing waves from the nodes 2 and *n* + 1 will meet at the node *i_m_*_2_ (see the arrows there) and then stop there. In this way, *i_m_*_1_ and *i_m_*_2_ will be the middle points of the smaller and larger loops, respectively, which gives *i_m_*_1_ = 2 + (*n* − 2)/2 = 16 and *i_m_*_2_ = *n* + 1 + [*N* − (*n* + 1) + 1 + (*i*_0_ − 2)]/2 = 95 for *n* = 30 and *N* = 150 and thus confirms the minimum points in both Fig. 2(b) and Fig. 3(a).

Then, we focus on the case of the panels (a), (c) and (d) of Fig. 2, i.e. the case of a heterogeneous pattern at the two branches of *f_b_* and *f_r_*. It will become Fig. 3(b) when the switch off is considered at *T*_0_ = 1600. In this case, a larger *f_r_* will contribute a larger *I_i_*_+1_ in Eq. (1) and thus will make the branch of *f_r_* easier to fire, resulting a match between the stimulus frequency *ω* and the refractory period of the *f_r_* branch. In this way, the firing frequency in the branch of *f_r_* is double of that in the branch of *f_b_*, which will result in a faster propagation of firing waves in the *f_r_* branch than that in the *f_b_* branch and thus causes the moving of *i_m_*_2_ in Fig. 2(a). This moving will reduce the opposite propagated firing waves from *i*_0_ to *i_m_*_2_ and finally reach a balanced ending position of *i_m_*_2_ in Fig. 2(a), (c) and (d).

**Figure 9:**
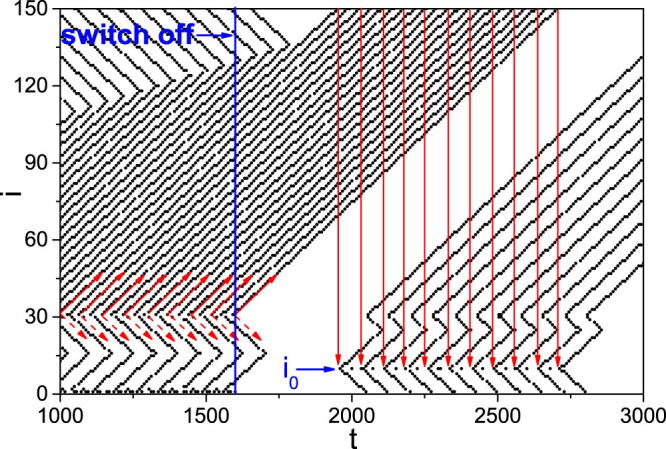
Amplification of Fig. 3(b) for 1000 < *t* < 3000 where the external stimulus is switched off at *t* = 1600. The solid arrows starting at *i* = 31 show the real propagation direction while the dashed arrows starting at *i* = 30 show the propagation direction supposed to be but not really occurred. The solid arrows from *i* = 150 to *i* = *i*_0_ represent the self-sustained propagation.

From Fig. 2(a), (c) and (d) we see that the ending position of *i_m_*_2_ depends on *T*_0_, which will determine the interval between two consecutive patterns in Fig. 3(b). Figure 9 shows the local amplification of Fig. 3(b) for 1000 < *t* < 3000 where the external stimulus is switched off at *t* = 1600. It is easy to see that before *t* = 1600, *i_m_*_2_ will move with *t* and the firing propagation along the branches of *f_r_* and *f_b_* at around the node *i* = 30 is exactly the same as in Fig. 3(a) in SI (see the “solid and dashed arrows” at around *i* = 30 and 31 in Fig. 9). After *t* = 1600, from Fig. 9 we see that the firing waves with solid arrows starting at *i* = 31 will be remained to form a sustained pattern which is marked by the solid arrows from *i* = 150 to *i* = *i*_0_, while other firing waves will be gradually canceled each other. Thus, the asymmetric propagation (represented by the “solid and dashed arrows” at around *i* = 30) induced by the difference between *f_r_* and *f_b_* guarantees the formation of LTM patterns. Let *l*_1_ be the firing number along the path 1 → *n* + 1 → *i_m_*_2_ and *l*_2_ be the firing number along the path 1 → 2 → *i*_0_ → *N* → *i_m_*_2_. Their difference *l*_1_ − *l*_2_ will increase with *T*_0_ until *l*_2_ reaches its minimum (see Fig. 2(a), (c) and (d)). Once the external stimulus is switched off, part of the firings *l*_1_ will gradually meet with the *l*_2_ firings and cancel each other. The net firings *l*_1_ − *l*_2_ will be remained in the network to form the LTM patterns.

We also notice from Fig. 9 that the pattern before *t* = 1600 is changed into a new one after *t* = 1600, where the previous source node 1 is replaced by a new source node *i*_0_ (see the “arrow with *i*_0_” there). This changing means that the LTM patterns are determined by the network topology of Fig. 1(c) such as the network parameters *N*, *n* and *i*_0_, see the flow chart Fig. 4 in SI for details. In sum, the network parameters will determine the size of pattern, the match between *f* and *ω* will determine the interval between two consecutive firings in a pattern, and *T*_0_ will determine the interval between two consecutive patterns of LTM. More complicated LTM patterns can be obtained if we add more small loops in Fig. 1(c).

## Discussion

Understanding the patterns of STM and LTM has been a challenging problem for a long time and is still at the core of cognitive and learning tasks. Although a lot of findings such as traveling waves have been achieved, the transition from STM to LTM and their diversity has not been paid enough attention so far. We agree that the core network model of Fig. 1(c) is a simplified motif of the real brain functional network, composed by a huge number of neurons, but it grasps the fundamental feature of STM and LTM, i.e. the diversity of patterns. It has been pointed out that memory is a kind of recall to the hidden patterns in the huge brain network where a small size of neurons will be activated for a specific event/picture^1^,^2^,^9^. In this sense, each memory involves only a finite neurons from the specific local part of the brain network, indicating that the core network model also has some practical meaning.

On the other hand, the core network of Fig. 1(c) can be easily grown into a large network by sequentially adding new nodes to the existing nodes, i.e. Fig. 1(b). That is, the core part can be considered as a pacemaker to generate the self-sustained patterns in the brain functional network with a large size. In this sense, we may even reduce the size of the core part, provided that it can generate the self-sustained patterns. To confirm the relationship between the whole network and its core part, Fig. 5 in SI shows the evolution of firings for the grown nodes and the core part, respectively, where the patterns are co-produced by both the core part and the surrounding part. We find that the core part and the surrounding part have the similar periodic behaviors when the stimulus is switched off, confirming that the patterns of LTM is controlled by the core part.

A realistic network may have multiple communities with different functions. Thus, the considered core network may be just the motif of one community, implying that it is possible to have many motifs in the brain functional network with a large size. The communication and interaction between the motifs will make the self-sustained firing patterns more plentiful, which results in the diversity of STM and LTM patterns and thus guarantee the powerful memory capacities of the brain.

Along the line of this study, a further key problem is how to implement the memory search and its related tasks by patterns recognition. This problem has been paid great attention in the earlier studies from the aspect of adaptive resonance theory (ART) by Grossberg^55^. The models of ART not only account for a wide range of normal human behavior, they also account for “abnormal” behaviors, such as hallucinations. In this sense, using the approach of patterns recognition to explain the diversity of memory behaviors in humans will definitely deepen our understanding to the STM/LTM phenomena and will be our next research direction.

In conclusions, we have presented a core network model to study the pattern formation of memories and the transition from STM to LTM, in contrast to the traditional experimental approaches. By this model we reveal that the diversity of STM and LTM patterns can be implemented by a small number of neurons, provided that three necessary conditions are satisfied. The first one is the heterogeneous synaptic conductance *f* in the steady state, which is essential for the LTM dynamics and includes both the case of distributed *f* and the case of two different constants of *f_b_* and *f_r_*. The match between different phase-lockings from the two branches in Fig. 1(c) will produce a net part of firings, which forms the LTM patterns. The second one is the topological parameters *N*, *n* and *i*_0_, which influence the size of pattern. The third one is the stimulus parameter *T*_0_, which determines the interval between two consecutive patterns. These findings suggest that the underlying mechanism of STM and LTM may be very simple, which is meaningful to the understanding of the brain network and can be expected in experiments of neuron circuits.

## Author Contributions

K.X. and Z.L. conceived the research project. K.X., X.Z., C.W. and Z.L. performed research. K.X. and Z.L. analyzed the results. Z.L. wrote the paper. All Authors reviewed the Manuscript.

## Supplementary Material

Supplementary Information A simplified memory network model based on pattern formations
