# Supplementary material for: A simplified memory network model based on pattern formations
Source: Sci Rep. 2014 Dec 19;4:7568. doi: 10.1038/srep07568 (PMC4271251; doi:10.1038/srep07568)
Supplement: Supplementary Information — A simplified memory network model based on pattern formations [file srep07568-s1.pdf]

A simplified memory network model based on pattern formations

Kesheng Xu, Xiyun Zhang, Chaoqing Wang and Zonghua Liu

1 Supplementary Figures

Figure 1(a) and (b) show two typical evolutionary processes for $\omega = 0.5$ and 1, respectively, where $f = 0.035$ and the stimulus is added to the source node 1 when $t \leq 1600$ and switched off when $t > 1600$. It is easy to see that the two patterns in both Fig. 1(a) and (b) are first generated at the source node 1, then propagated to other nodes through its three neighboring nodes $2, 30$ and 31, and finally stabilized until $t = 1600$, indicating that the stimulus has been detected and transmitted. We also notice that for each neuron, there is a gap between any two consecutive firings, indicating that the corresponding stimulus in the gap is not detected. [ht]

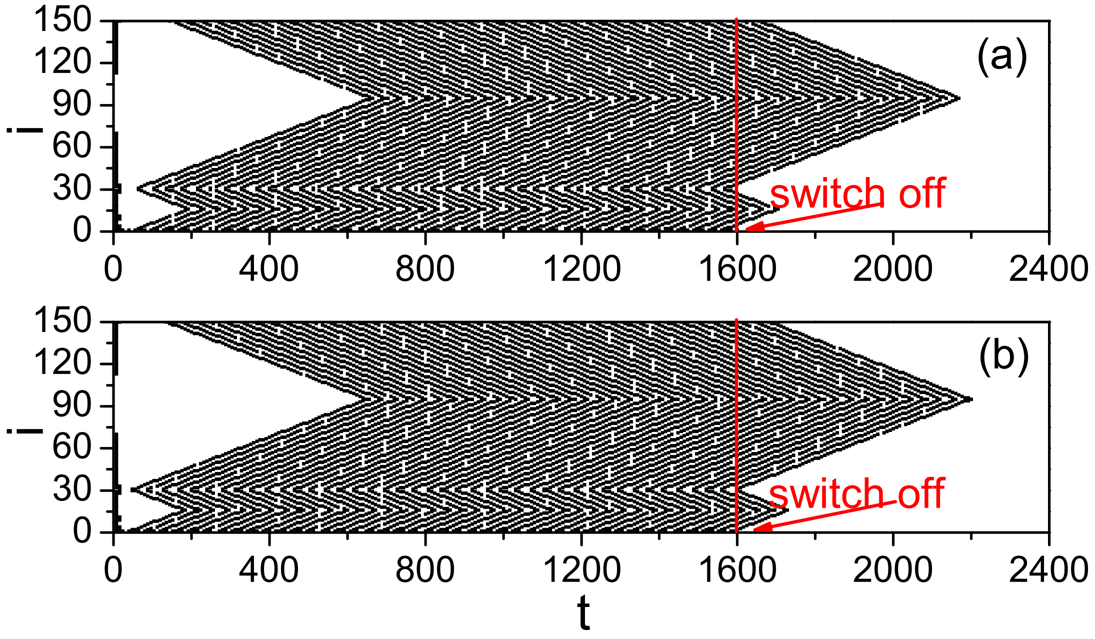

(a)
(b)
switch off
switch off
i
t

Figure 2 shows four typical patterns for $i_0 = 10$ and $\omega = 0.5$ where (a) represents the case of $T_0 = 400$, (b) the case of $T_0 = 800$, (c) the case of $T_0 = 1200$, and (d) the case of $T_0 = 1600$. A common feature in Fig. 2(a)-(d) is that the patterns in each panel are regular or periodic. [ht]

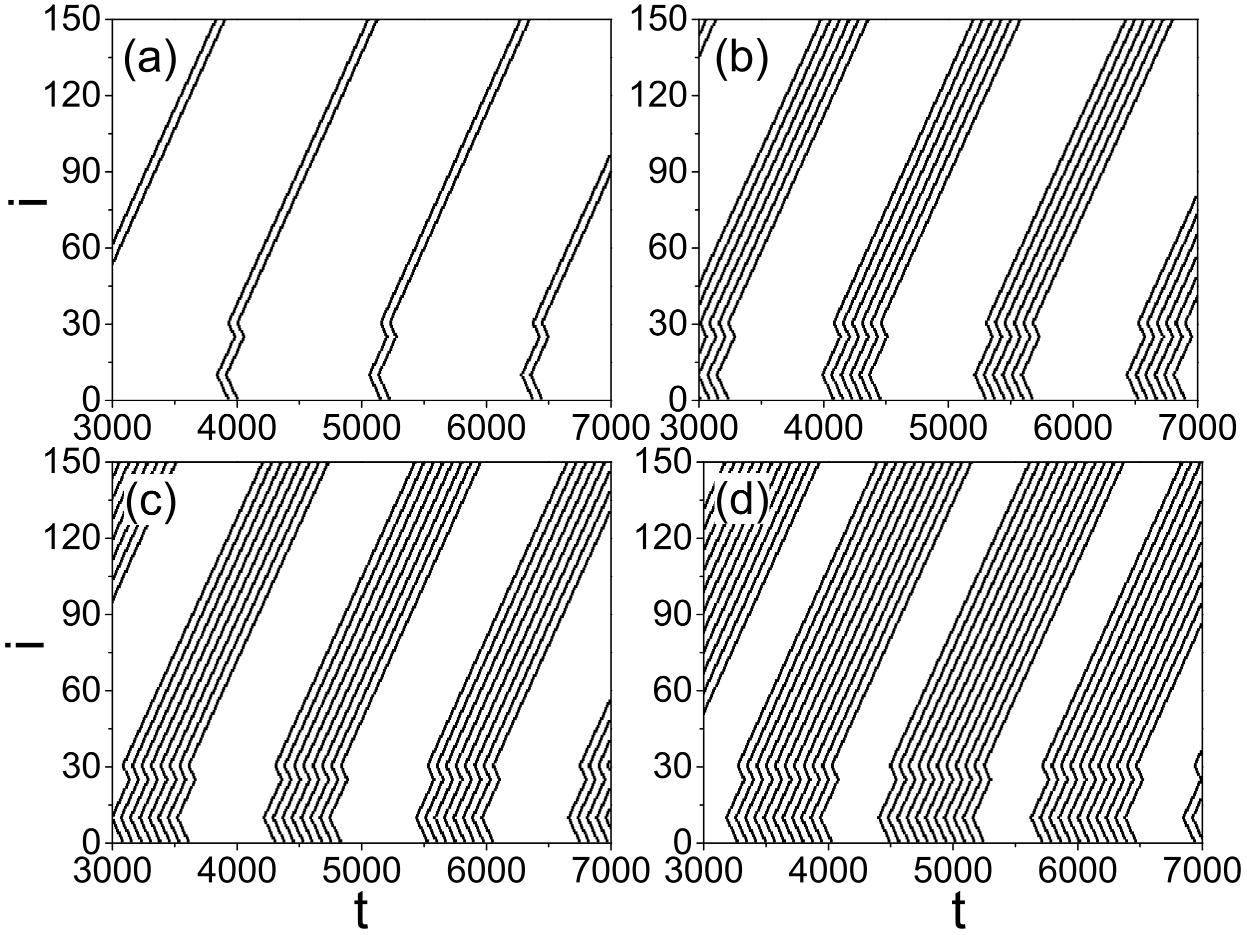

It is necessary to understand how f_b and f_r influence the patterns of STM and LTM. For this purpose, we show the local amplification of Fig. 2(a) and (b) in main text in Fig. 3(a) and (b), respectively. From Fig. 3(a) and (b) we see that the node 1 fires 13 times in (a) and 19 times in (b), indicating that the larger $\omega = 0.75$ in (b) will generate more firings than that in (a) with smaller $\omega = 0.5$. Notice that the signal period is $2\pi/0.5 \approx 12.5$ in Fig. 3(a) and $2\pi/0.75 \approx 8.4$ in Fig. 3(b). Thus, the number of periods is $500/12.5 = 40$ in Fig. 3(a) and $500/8.4 \approx 60$ in Fig. 3(b), indicating that there is a firing at node 1 in every three periods of external signal in both Fig. 3 (a) and (b). That is, because of the existence of refractory status of neurons, some periods of stimulus will not produce a firing.

From Fig. 3(a) we see that most of the firings generated at node 1 can be propagated to the branch of f_r ("up solid arrows") but only half of them can be propagated to the branch of f_b, i.e. half of them is lost ("down dashed arrows"). However, from Fig. 3(b) we see that only half of the firings generated at node 1 can be propagated to both the branch of f_r and the branch of f_b, i.e. half of them is lost ("up and down dashed arrows"). This difference tells us that the propagation of firings is determined by both the frequency ω and the difference between f_r and f_b.

[ht]

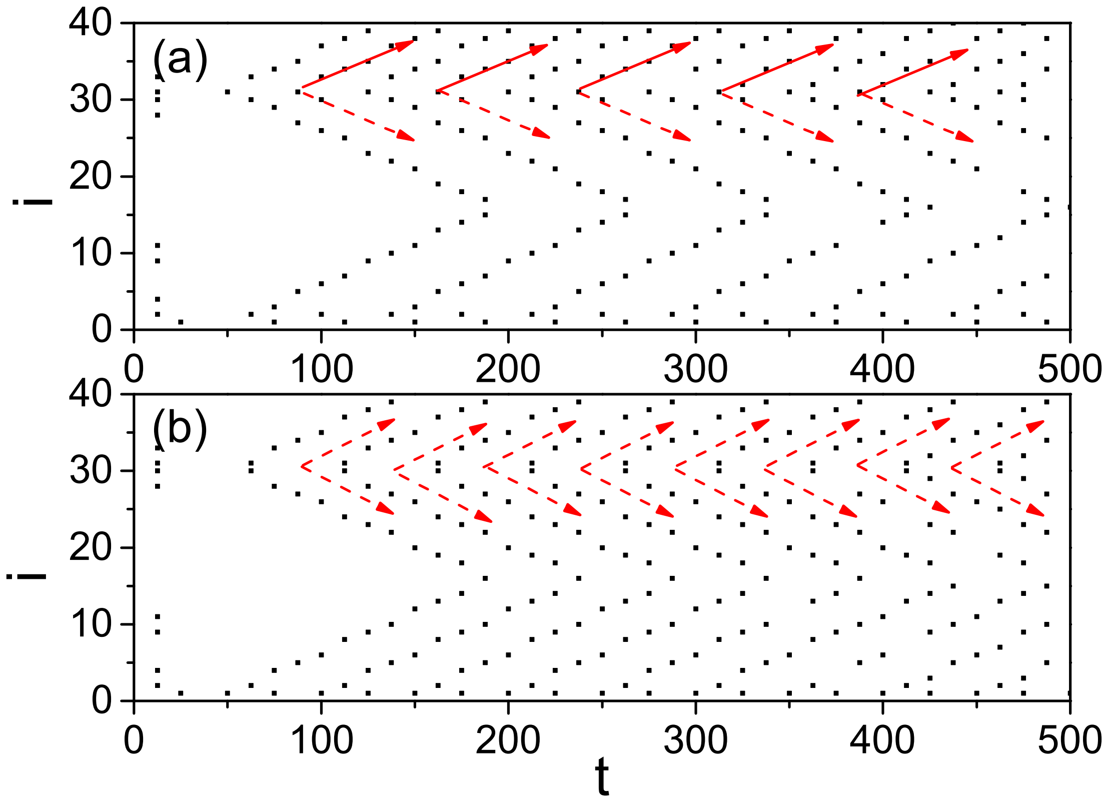

(a)
(b)
i
t
0
10
20
30
40
0
100
200
300
400
500

To make it clear, Fig. 4 shows the flow chart of the pattern of LTM. From this figure we see that in contrast to Fig. 1(c) in main text, there is no i_{m2} in Fig. 4 and the propagation direction of the middle branch is inverse to that of Fig. 1(c) in main text. Thus, the formed LTM patterns are in fact a series of traveling waves along the bigger loop and each periodic behavior in Fig. 3(b) in main text comes from one round of running on the loop. The firings on the small loop are dependent and just make the LTM patterns be complicated. More complicated LTM patterns can be obtained if we add more small loops in Fig. 1(c) in main text. [ht]

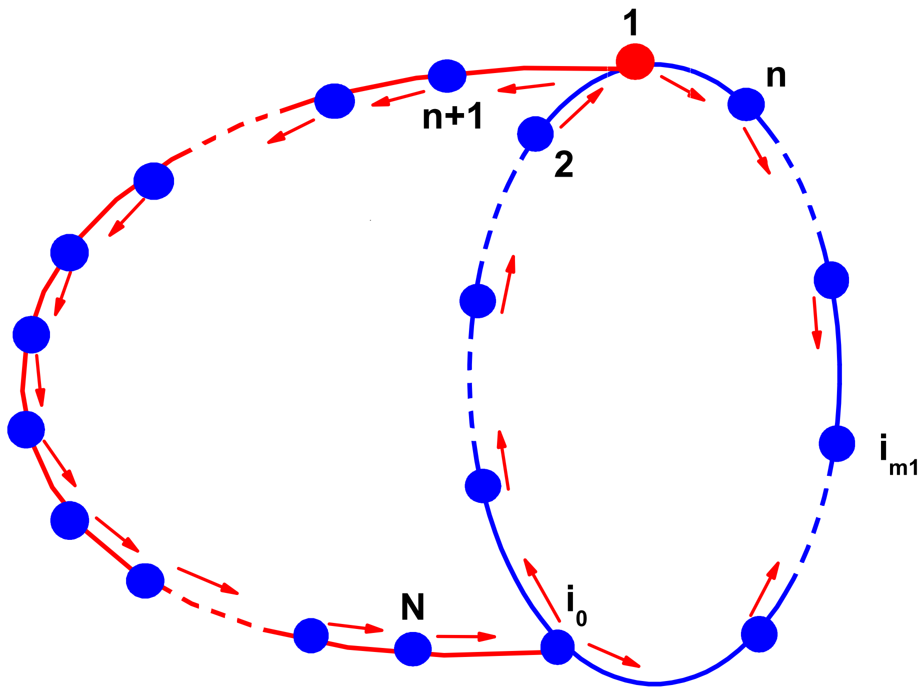

We here discuss the influence of T_0. From Fig. 4(b) and (c) in main text we find that depending on the match between ω and i_0, the stabilized S_p may have two fates, i.e. zero or nonzero. From Fig. 4(a) in main text we see that once S_p is zero, it will be kept for increasing T_0. While for the case of nonzero S_p, S_p increases with T_0 until a maximum. This maximum S_p is determined by the length of the bigger loop in Fig. 4. Because of the existence of refractory status, a neuron will take a time interval Δt to rest after its firing. A persistent stimulus will make the source node 1 continuously generate firings after each resting time Δt and these firings will be consecutively propagated to other nodes. As we have noticed in Figs. 2 and 3 in main text, the moving of the meeting place i_{m2} is the key reason to form LTM patterns. Because of this moving of i_{m2}, the firing number l_1 along the path $1 \to n+1 \to i_{m2}$ will be greater than the firing number l_2 along the path $1 \to 2 \to i_0 \to N \to i_{m2}$ and their difference $l_1 - l_2$ will increase with T_0 until l_2 reaches its minimum (see Fig. 2(a), (c) and (d) in main text). Once the external stimulus is switched off, part of the firings l_1 will gradually meet with the l_2 firings and cancel each other. The net firings $l_1 - l_2$ will be remained in the network and follow the flow chart of Fig. 4 to form LTM patterns. Longer T_0 will make larger $l_1 - l_2$, confirming the diversity of patterns in Fig. 2.

In numerical simulations, we find that the observed results are robust to the network size. Thus, we here reduce the size of the core network to $N = 50$ and $n = 20$ and choose the other parameters as $\omega = 0.5, i_0 = 10$ and $T_0 = 1500$ for the core part. We let the surrounding green part in Fig. 1(b) in main text have 294 nodes with f_r. Fig. 5 shows the patterns before and after switching off the stimulus at $t = T_0$. It is easy to see that before $t = T_0$, the patterns are gradually become sparser from the core part to the surrounding part, indicating the effect of decaying with the distance to the core. By the red line we also notice that there is a delay in the propagation of traveling waves and the delay increase with the distance to the core. However, after $t = T_0$, the patterns have the similar periodic behaviors from the core to the surrounding parts, confirming that the network's LTM patterns are controlled by the core part.

[ht]

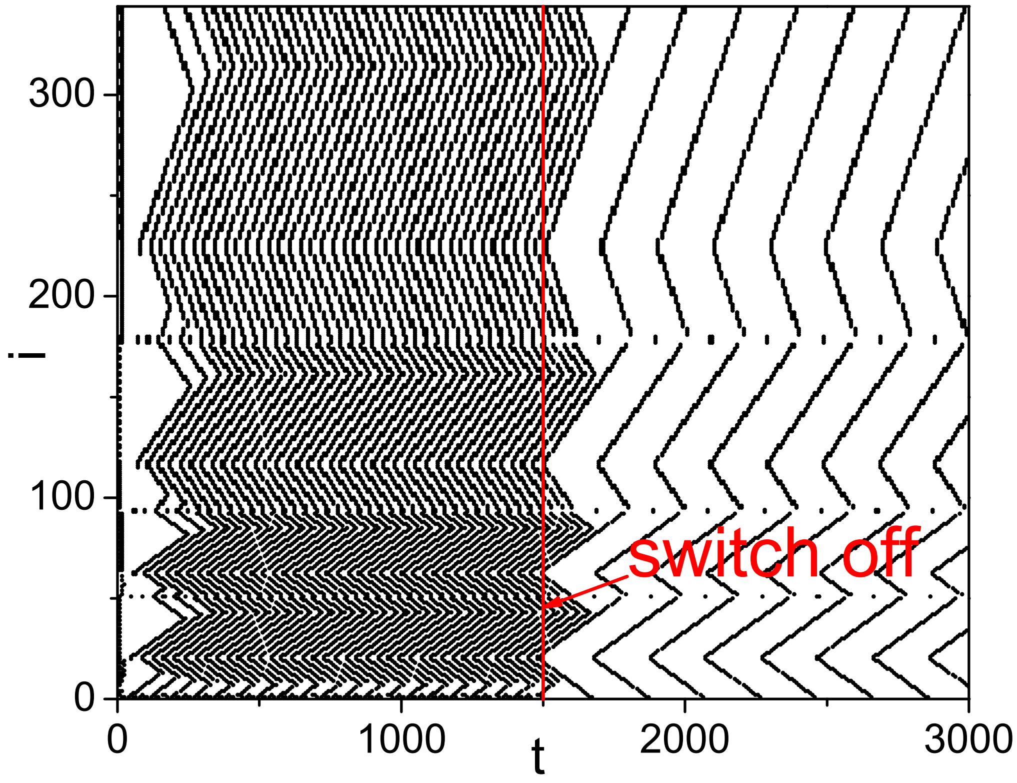

300
200
100
0
i
0
1000
2000
3000
t
switch off

Figure 1 (Color online.) **Case of identical f to all neurons**. Case of identical f to all the nodes with $f = 0.035$ where $i_0 = 10$ and the stimulus is added to the source node 1 and switched off at $t = T_0 = 1600$. (a) and (b) represent the cases of $\omega = 0.5$ and 1, respectively.

Figure 2 (Color online.) **Stabilized patterns for different stimulus T_0.** Patterns for different stimulus T_0 with $i_0 = 10, \omega = 0.5$. (a) represents the case of $T_0 = 400$, (b) $T_0 = 800$, (c) $T_0 = 1200$, and (d) $T_0 = 1600$.

Figure 3 (Color online.) **Local amplification of Fig. 2(a) and (b) in main text**. Amplification of Fig. 2 in main text where the panels (a) and (b) correspond to Fig. 2(a) and (b) in main text, respectively. The solid arrows show the real propagation direction while the dashed arrows show the propagation direction supposed to be but not really occurred.

Figure 4 (Color online.) **Flow chart of LTM patterns after switching off the stimulus**. Flow chart of LTM patterns after switching off the stimulus, where the arrows represent the direction of firing propagation, which is different from that in Fig. 1(b) in main text.

Figure 5 (Color online.) **Evolution of the firing patterns in the grown network**. The network consists of 344 nodes with 50 nodes in the core part and 294 nodes in the surrounding part. The surrounding nodes are grown by the approach of Cayley tree from the boundary nodes of the core part and have the conductance f_r. The stimulus is switched off at $T_0 = 1500$.
